# A comparative analysis of platelet-rich plasma alone versus combined with extracorporeal shockwave therapy in athletes with patellar tendinopathy and knee pain: a randomized controlled trial

**DOI:** 10.1186/s43019-024-00252-3

**Published:** 2024-12-17

**Authors:** Shun-Wun Jhan, Kuan-Ting Wu, Wen-Yi Chou, Po-Cheng Chen, Ching-Jen Wang, Wen-Chiung Huang, Jai-Hong Cheng

**Affiliations:** 1 Department of Orthopedic Surgery, Center for Shockwave Medicine and Tissue Engineering, Kaohsiung Chang Gung Memorial Hospital and Chang Gung University College of Medicine, Kaohsiung, 833 Taiwan; 2 Center for Shockwave Medicine and Tissue Engineering, Medical Research, Kaohsiung Chang Gung Memorial Hospital and Chang Gung University College of Medicine, Kaohsiung, 833 Taiwan; 3 Department of Physical Medicine and Rehabilitation, Kaohsiung Chang Gung Memorial Hospital, 123 Dapi Road, Niao Sung District, Kaohsiung City, 833 Taiwan

**Keywords:** Patellar tendinopathy, Platelet-rich plasma, Extracorporeal shockwave therapy, Combination therapy, Functional improvement

## Abstract

**Background:**

Patellar tendinopathy, also known as jumper’s knee, can significantly impact the quality of daily life for patients due to the associated pain. A randomized controlled trial was investigated the clinical, sonographic, and serum cytokine markers in patellar tendinopathy of athletes following platelet-rich plasma (PRP) or PRP with extracorporeal shockwave therapy (ESWT) treatments. Our aims to investigate and compare therapeutic effects of PRP versus a combination of PRP with ESWT for treating patellar tendinopathy.

**Methods:**

A total of 33 athletes with patellar tendinopathy were randomized into two groups. PRP + Sham (PS) group received intraarticular injection of autologous PRP (5 mL) once and sham ESWT. PRP + ESWT (PE) group received intraarticular injection of autologous PRP once and after 1 week ESWT (0.2 mJ/mm^2^ energy flux density, 1350 impulses, 4 Hz) once. All patients were followed up for 1 year.

**Results:**

Autologous PRP injection and its combination with ESWT are both effective treatments for chronic patellar tendinopathy in athletes. PRP combined with ESWT resulted in faster reduction of knee pain than PRP alone at the 1-month follow-up. Serum IL-33 showed no significant difference at the 12-month follow-up. Levels of interleukin (IL)-6, IL-15, and IL-17 increased at the 12-month follow-up, potentially due to the additional training. However, the athletes did not report any discomfort or injuries, and no abnormalities were detected by ultrasonography after study. We demonstrated improvements in pain and functional scores, as well as knee injury protection in athletes, following 12 months of PRP and PRP with ESWT treatments.

**Conclusions:**

The study analyzed the therapeutic effect of PRP injection alone and combining PRP injection with ESWT for chronic patellar tendinopathy. Our results showed that combined treatment can facilitate the pain relief early than PRP alone and is a safety treatment modality. No adverse effect was noted in our study.

*Trial registration* Research registry and the registration number is researchregistry9518. Registered 14 September 2023. https://www.researchregistry.com/browse-the-registry#home/registrationdetails/650263e4f549fd00282a338c/. The level of evidence is level II.

## Background

Patellar tendinopathy is a common cause of knee pain in the elite athletes. It is also known as jumper’s knee because of its relationship with jumping sports. Prevalence of patellar tendinopathy had been reported 45% in volleyball players and 32% in basketball players [1]. However, repetitive stresses in the knee extensor mechanism can also lead to patellar tendinopathy. Among soccer players, 2.4% have characteristic symptoms of patellar tendinopathy [2].

The classic symptom of patellar tendinopathy is pain over proximal or distal part of patellar tendon. The pain is insidious and activity related. The physical examination of patellar tendinopathy included tenderness over proximal or distal part of patellar tendon and decline squat test, which performed by single leg squat when knee was in 30° flexion [3]. Noninvasive conservative treatment for patellar tendinopathy included cessation of the aggravating activities, eccentric exercise, use of nonsteroidal antiinflammatory drugs (non-steroidal antiinflammatory drugs, NSAIDs) and use of a patellar strap [4]. In most patients, above therapies have satisfactory result. However, in some high-level professional athletes or patients with chronic patellar tendinopathy, other more aggressive therapeutic strategies should be considered [5]. Open or arthroscopic surgery are alternative treatment options. Arthroscopic patellar release has been reported with good results [6]. Arthroscopic debridement of adipose tissue of the Hoffa’s body and abnormal patellar tendon also provided significant improvements in symptoms and function [7]. However, surgery is more invasive and associated with risks and complications. In addition, some athletes may be unable to undergo surgery due to being in the midst of training or competitive season. Regenerative therapies should be considered for those athletes with chronic patellar tendinopathy. Extracorporeal shockwave therapy (ESWT) and platelet-rich plasma (PRP) injection had been reported with convinced results [4, 5, 8].

PRP is widely used for its regenerative potential due to the high concentration of growth factors that promote healing. These growth factors, including platelet-derived growth factor (PDGF), transforming growth factor-beta (TGF-β), and vascular endothelial growth factor (VEGF), enhance tissue repair by stimulating cellular proliferation, angiogenesis, and extracellular matrix synthesis [9]. In the context of tendinopathy, PRP can modulate inflammation and promote tendon healing through these biological processes, making it a promising treatment option for chronic tendinopathy cases where conservative treatments have failed [10].

ESWT has been reported effective in relieving pain and improving function in patients with tendinopathy of shoulder, elbow, knee, and heel. Biomechanically, ESWT had effects including neovascularization, antiinflammation, and tissue regeneration. Shockwave can stimulate the ingrowth of neovascularization at the tendon–bone junction [11, 12]. Clinically, Wang et al. also reported that ESWT is an effective and safe treatment for patient with chronic patellar tendinopathy [13].

The use of ESWT is based on its mechanical and biological effects on tendon healing. ESWT can modulate cell signaling pathways, reduce local inflammation, and promote neovascularization and collagen synthesis, which are critical for tendon repair. Moreover, ESWT has the advantage of being noninvasive and relatively free of adverse effects, making it suitable for athletes who require minimal downtime [14].

However, there are still some patients with chronic patellar tendinopathy who have persistent and aggravated symptoms after PRP or ESWT therapies alone [15, 16]. The therapeutic effect of single PRP injection in treating chronic patellar tendinopathy also diminished over time [17, 18]. We are interested in investigating whether a combined treatment regimen involving a single PRP injection and multiple sessions of ESWT application could yield enhanced therapeutic effect, particularly for those athletes actively engaging in training or competitive seasons.

## Methods

### Study design

The study is a single-center, double-blind randomized clinical trial and approved by the Institutional Review Board of the hospital and the work has been reported in line with the CONSORT checklist [19]. In addition, the clinical trial was registered from Research Registry (https://www.researchregistry.com/).

Initially, 36 athletes with chronic patellar tendinopathy were screened for eligibility in this randomized clinical trial. Each athlete underwent a thorough assessment, including clinical history, physical examination, and imaging [ultrasonography or magnetic resonance imaging (MRI)] to confirm the diagnosis. To ensure homogeneity, inclusion criteria required participants to be 15 years or older, report a persistent pain score of 5 or higher on a 0–10 visual analogue scale (VAS) for at least 3 months, and provide informed consent. For athletes aged 15–20, parental consent was also obtained. Exclusion criteria included prior injections into the patellar tendon within the past 6 weeks, coexisting knee pathologies (e.g., ligament injuries, meniscal tears), systemic conditions affecting healing (e.g., uncontrolled diabetes), use of medications affecting platelet function (e.g., corticosteroids), and pregnancy. After applying these criteria, 3 athletes were excluded due to recent injections, leaving a final cohort of 33 participants; 16 were randomized to the PRP + Sham (PS) group and 17 to the PRP + ESWT (PE) group. This selection process ensured a well-defined population for evaluating treatment efficacy. Control group (PS group) includes 16 athletes who were treated with combination of sham-shockwave treatment and autogenous PRP injection. Study group (PE group) includes 17 athletes received combination of focused ESWT and autogenous PRP injection. The sample size was calculated using G*Power software (version 3.1.9.7) with a post hoc power analysis (two-tailed test). Given the primary outcome measure (VAS score) and expected effect size based on prior studies [13, 20], the analysis was conducted to ensure sufficient statistical power for detecting a clinically meaningful difference. On the basis of prior studies on ESWT in similar populations, we used an estimated standard deviation of 1.8 points for the VAS score [21]. We set the significance level (*α*) at 0.05, aiming for a power of 85% (1 − *β* = 0.85), which is considered adequate for this type of clinical investigation. With these parameters, the software yielded a noncentrality parameter of 3.121, a critical *t*-value of 2.040, degrees of freedom (*df*) of 31, and a calculated power of 0.856. On the basis of these results, a sample size of 33 participants (16 in the control group and 17 in the treatment group) was determined to be sufficient.

### Randomization and blinding

We employed computer-generated randomization to allocate athletes into different groups. The treatment group assignments were placed in sealed envelopes. On the day of treatment, medical assistant will open the sealed and explain the treatment protocol to other medical staff responsible for preparing PRP and performing ESWT application.

Throughout the entire treatment process, both orthopedic doctors, rehabilitation physicians, and patients remained blinded to their respective assignments. The medical assistant who performed the ESWT application was not blinded. The orthopedic doctors would record the evaluation parameters including VAS, Victorian Institute of Sport Assessment-Patella (VISA-P), and Modified Blazina scale (MBS) at every timepoint. The rehabilitation physicians wound perform the ultrasonography.

None of our patients declined treatment or crossed over into another group due to dissatisfaction. All athletes remained blinded until final follow-up at 1 year. We took precautions to ensure that any language in billing and written communications that unblind the patient was removed.

### Autologous PRP injection

One week before ESWT, all athletes in both groups receive one autologous PRP injection at the lesion site. The PRP processing is performed using the Arthrex ACP Double-Syringe System. This system uses a dual spin system and is a fully enclosed system that maintains sterility throughout the entire process. The 15 mL of whole blood were collected and then used the Arthrex ACP Double-Syringe System to collect the autologous PRP. This system generally yields a platelet concentration approximately 2–3 times the baseline level of whole blood while reducing the concentration of white blood cells and inflammatory factors. Local anesthetic agent does not need before PRP injection. The skin of the injection site is prepped and draped, then approximately 5 mL of liquid autologous PRP, with platelet concentrations in the range of 300,000–1,200,000 platelets/μL, is injected in sterile condition using a 21G needle with ultrasound guidance. Athletes do not receive non-steroidal antiinflammatory drugs (NSAIDs) for 2 weeks after PRP injection.

### Shockwave application

The source of shockwave is from a DUOLITH SD1 device (Storz Medical AG, Tägerwilen, Switzerland). ESWT application consisted of 1350 impulses of shockwaves at energy level 1 (equivalent to 0.2 mJ/mm^2^ energy flux density, 4 Hz) in a single session as outpatients with no local or regional anesthesia. The maximal tender point was elicited by palpation and the location of ESWT applied was focused with the laser guide of shockwave machine. The shockwave tube is in contact with skin after surgical lubricant applied. After shockwave procedure, the treatment area was evaluated for redness, swelling, or other abnormal skin lesions. Post-therapeutic education included ice packing for treatment site and allowed light activity. We do not prohibit training programs or competitive games after treatment if patients can tolerate their symptoms.

The sham-shockwave treatment (placebo) procedure for the control group is nearly the same as the ESWT treatment and is administered with the same device, in a single session, using 1350 pulses in a frequency of 4 Hz and an energy level 1. Transmission gel is applied on the skin of the participants, but no focusing pad on the skin of the participants. In this way, shockwaves are no conducted to tissue.

### Evaluation parameters of the visual analogue scale, functional scores, and ultrasonography

All athletes accepted examinations, including visual analogue scale (VAS), functional score (VISA-P and MBS), and ultrasonography, before PRP injection. The blood analysis, VAS, and functional scores were done repeatedly at 1, 3, 6, and 12 months. The ultrasonography was scheduled at 6 and 12 months. Any adverse events will be documented at each follow-up visit.

The clinical examinations included VAS pain score, VISA-P and MBS. The VAS is one of the pain-rating scale, in which 0 means no pain and 10 means extreme pain when walking, upstairs and downstairs. The VISA-P is a questionnaire that assesses symptoms, function, and sports activity of patients with patellar tendinopathy. The VISA-P questionnaire, including 8 questions, had max score 100 for asymptomatic patient and theoretical minimum score 0. MBS is a staging system to evaluate sports activity. Stage 0 means no pain and stage 5 means unable to participate in sport at any level.

The subjective patient outcome for return to sports (SPORTS) scale is a specialized scoring system developed to evaluate an individual’s return to sports, performance level, and any residual impairments related to sports participation [22]. It categorizes performance on a 5-point scale. A score of 10 is given to athletes who can participate in their sport at the same level of effort and performance as before the injury, with no pain. Those experiencing mild pain receive a score of 9. Athletes maintaining the same effort but showing reduced performance compared with preinjury levels are scored a 6. If both effort and performance are lower than before the injury, the score is 3. Athletes unable to resume the same sport receive a score of 0. Previous studies have demonstrated the SPORTS scale’s validity and reliability in assessing functional outcomes and quantifying return to sports [23].

The ultrasonography of the affected knee was done by a specialist before the treatment for diagnosis and repeated at 3 and 6 months. The examined locations included proximal and distal part of patellar tendon. The evaluated parameters included thickness and elasticity of patellar tendon.

### Enzyme-linked immunosorbent assay

About 10 mL of blood was collected from athletes and the serum was stored at −80 °C for use. Serum sample evaluation of the athletes using serum enzyme-linked immunosorbent assay (ELISA) and clinical examinations were carried out before treatment (week 0) and after at 1, 3, 6, and 12 months. The serum makers of neovascularization, inflammation, and tissue regeneration, including IL (interleukin)-6, IL-15, IL-17, IL-33, and vascular endothelial growth factor (VEGF)-A (R&D Systems®, USA) were collected for analysis.

### Statistical analysis

SPSS ver. 26.0 (SPSS Inc., Chicago, IL, USA) was used in statistical analysis. The data were expressed as mean ± SD. Independent *t*-test was used to compare the difference of age between two groups. Chi-squared test was used to compare the difference of gender and side between two groups. Generalized estimating equations was used to compare the differences of VAS, VISA-P, MBS, SPORTS scale, cytokines, and ultrasonographic findings between the two groups (PS and PE). False discovery rate (FDR) was used for probabilities correction. The *p*-values less than 0.05 were accepted as statistically significant.

## Results

### Comparison of the VAS and functional scores before and after treatments

**Fig. 1:**
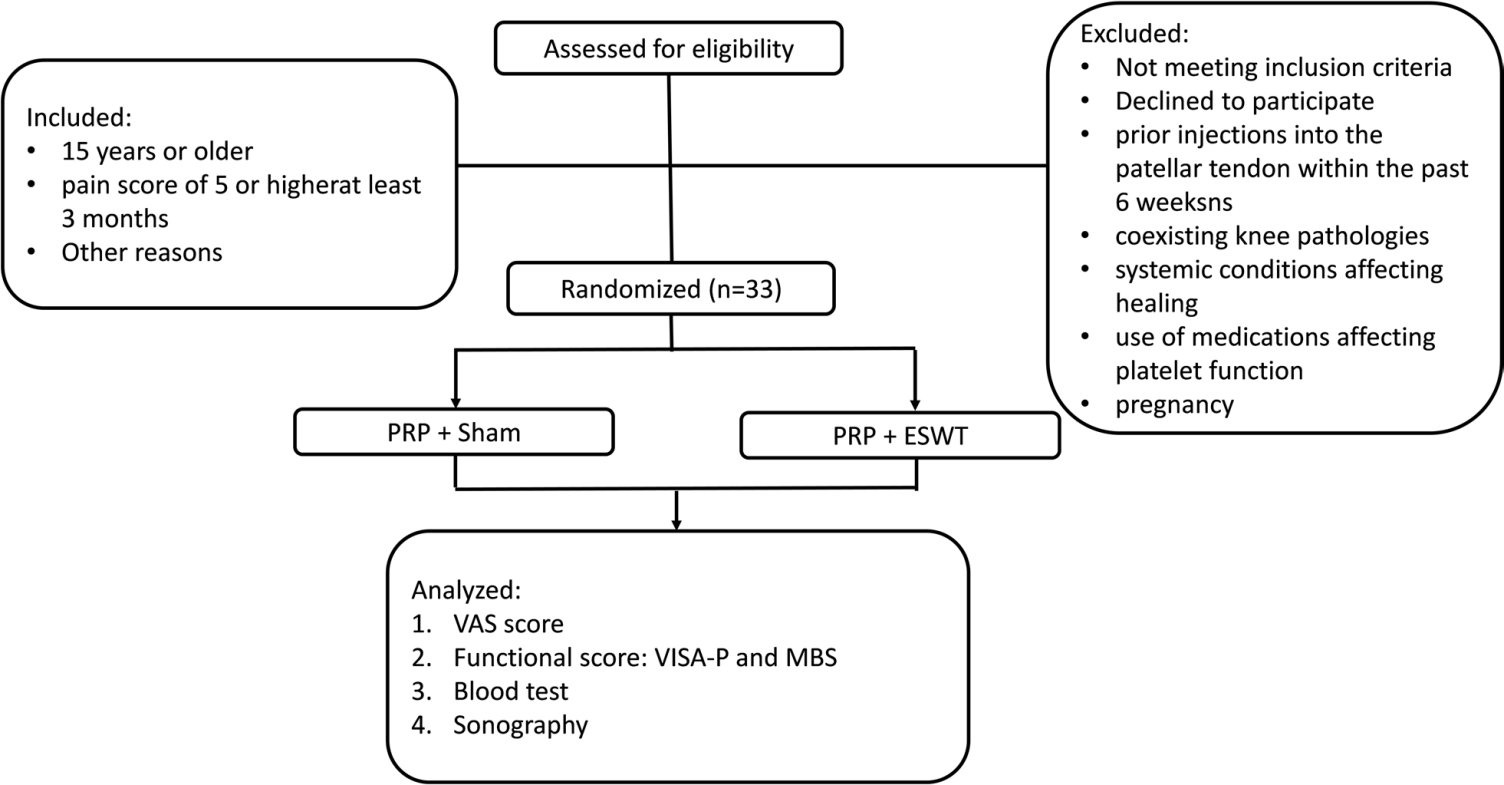
CONSORT flow diagram are presented. *PRP* platelet-rich plasma, *ESWT* extracorporeal shockwave therapy, *VAS* visual analogue scale, *VISA-P* Victorian Institute of Sport Assessment-Patella, *MBS* modified Blazina scale

The CONSORT flow diagram is shown in Fig. 1. A total of 33 athletes were randomized into two groups and the demographic data are presented in Table 1. Clinical outcomes and analysis before and after treatment were presented in Table 2. Before treatment, there were no significant differences in VAS, VISA-P, and MBS between groups. Both groups, PS and PE, had significant pain relief after treatments (Table 2, *p* < 0.05). In functional score, both groups also had significant improvement in VISA-P and MBS after treatments (Table 2, *p* < 0.05). Comparing the data of VAS, VISA-P, and MBS improvements between two groups, there were no significant differences in all three parameters.

**Table 1:** Demographic data

	PS	PE	*p*-Value
Numbers of patients	16	17	
Numbers of knees	16	17	
Average age, years old	16.8 ± 1.7 (15–22)	17.7 ± 2.5 (15–25)	0.417
Gender (male/female)	10/6	12/5	0.622
Side (right/left)	7/9	10/7	0.387
Sports			
Football	2		
Swimming		1	
Jogging	3	1	
Tug of war	2		
Baseball	1		
Weightlifting	5	10	
Athletics		1	
Badminton	1	2	
Volleyball	2	3

**Table 2:** Results of pain scores and functional outcomes and SPORTS scale between two groups before and after treatment

	PS (PRP + Sham)	PE (PRP + ESWT)	*p-value
VAS score			
Before treatment	7.31 ± 0.87	8.00 ± 1.06	> 0.05
After treatment	0.75 ± 1.57	1.65 ± 2.00	> 0.05
*p*-value^#^	< 0.001	< 0.001	
VISA-P			
Before treatment	56.00 ± 15.03	59.12 ± 9.05	> 0.05
After treatment	94.31 ± 10.89	87.18 ± 11.28	> 0.05
*p*-value^#^	< 0.001	< 0.001	
MBS			
Before treatment	2.88 ± 0.81	2.94 ± 0.24	> 0.05
After treatment	0.38 ± 0.62	0.94 ± 0.83	> 0.05
*p*-value^#^	< 0.001	< 0.001	
SPORTS scale			
Before treatment	4 ± 0.5	3 ± 0.2	> 0.05
After treatment	10 ± 0.3	9 ± 0.5	> 0.05
*p*-value^#^	< 0.001	< 0.001	

*The *p*-values less than 0.05 are accepted as statistically significant as compared between the PS and PE groups

^#^The *p*-values less than 0.05 were accepted as statistically significant as compared with the data before treatment (Pre)

Before the treatment, the average SPORTS scale scores were 4 ± 0.5 in the PS group and 3 ± 0.2 in the PE group. By the final follow-up, these scores had increased to 10 ± 0.3 and 9 ± 0.5, respectively, indicating significant improvements in both groups (Table 1, *p* < 0.05). In the PS group, all patients except one, who had a SPORTS scale score of 6, achieved scores greater than 9 at the final follow-up. In the PE group, three patients had scores below 6, while the remaining participants had scores above 9 at the final follow-up.

### Time chasing and tendency of VAS, VISA-P, and MBS scores after PRP and PRP combined with ESWT

**Fig. 2:**
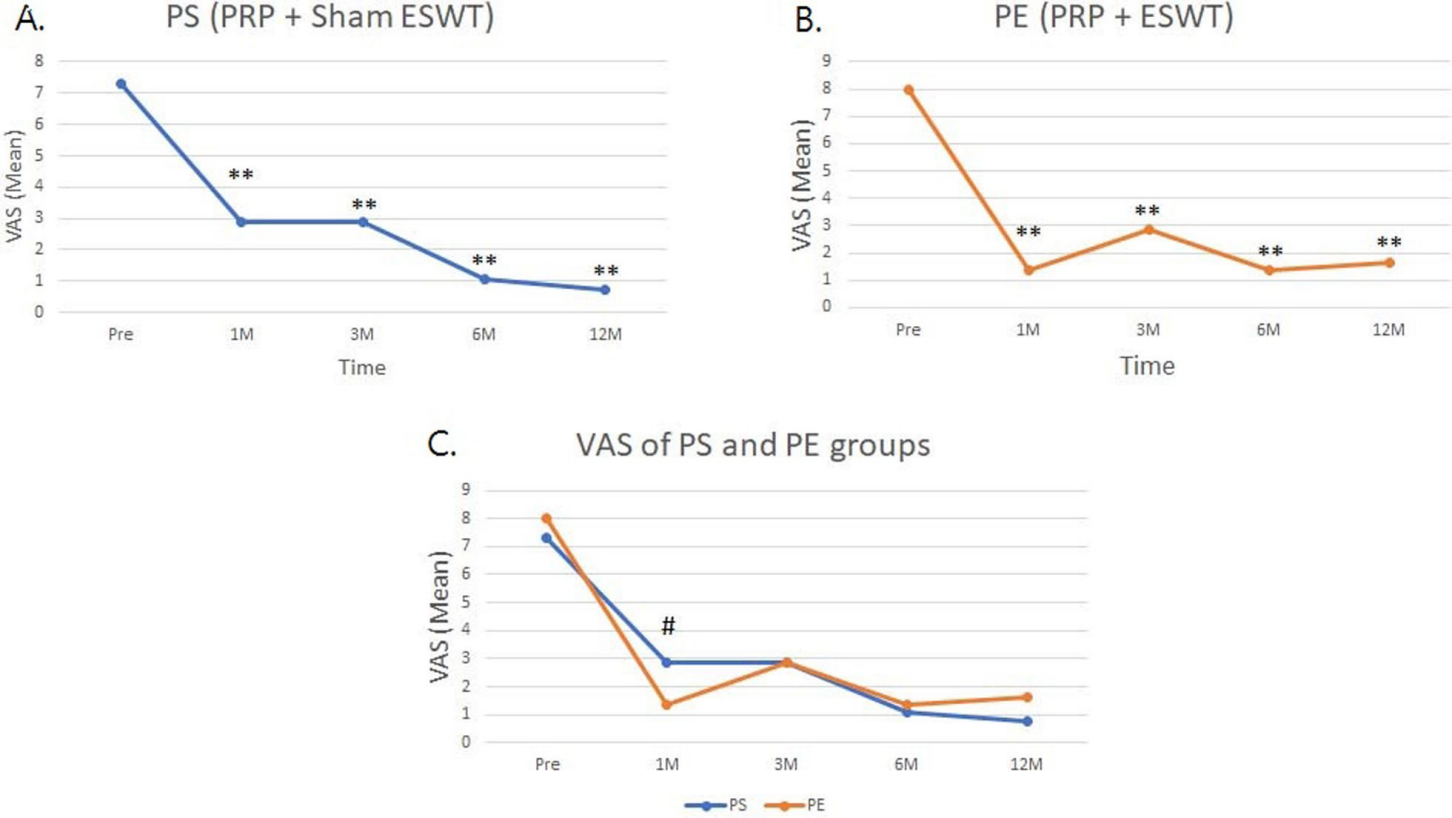
Trends of VAS score are improvement during the treatment course at 1, 3, 6, and 12 months (M). VAS is illustrated in **A** PS group, **B** PE group, and **C** comparison of VAS score between PS and PE groups panels. PRP + Sham: PS group. PRP + ESWT: PE group. The *p-value < 0.05, **p-value < 0.01 are as compared with the data before treatment (Pre) in **A** and **B**. The ^#^*p*-value < 0.05 means significant differences of pain relief between two groups at any timepoint in **C**

To observe the effects of both treatments over time, VAS scores significantly decreased and remained reduced up to the 1-year follow-up after treatments (Fig. 2A, B). Additionally, the PE group experienced more pain relief than the PS group at the 1-month follow-up (Fig. 2C, p < 0.05). These results demonstrate that PRP combined with ESWT can reduce pain more quickly than PRP alone.

**Fig. 3:**
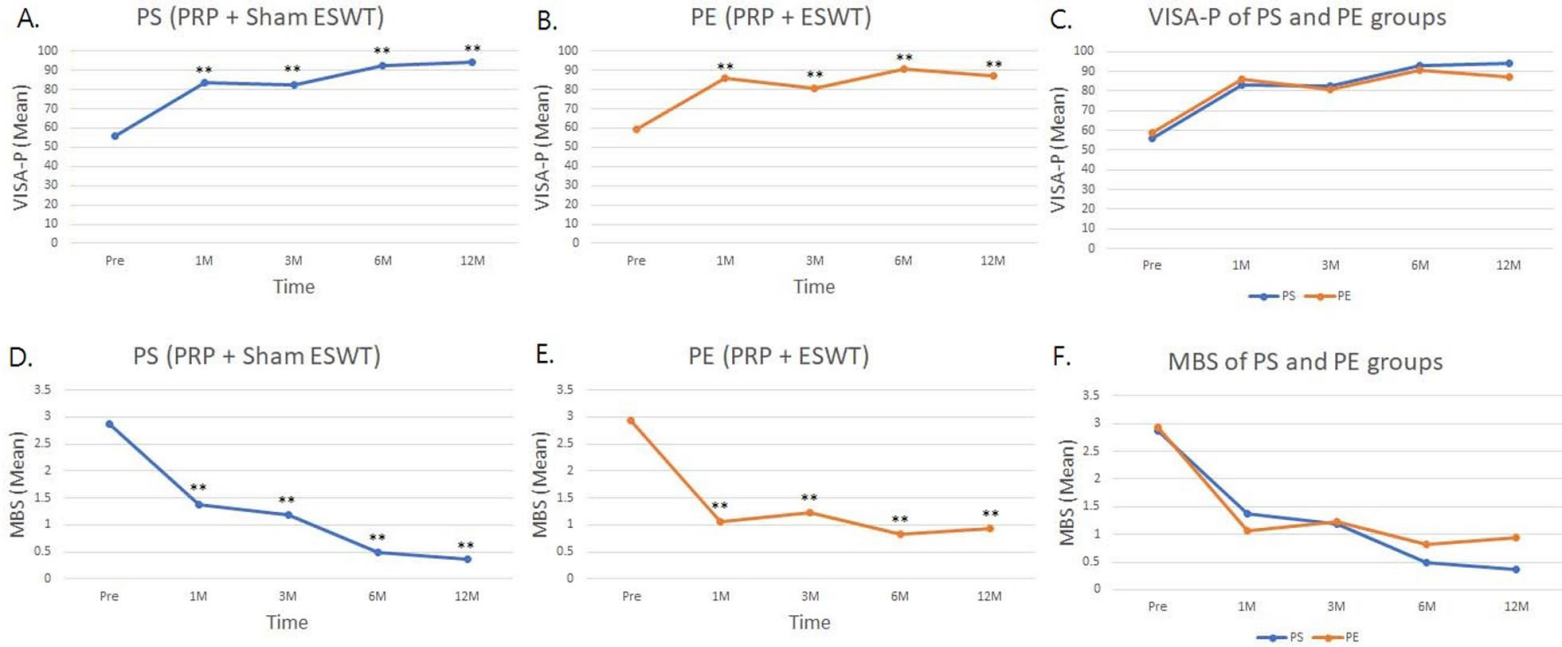
The trends of VISA-P and MBS are improvement during the treatment course at 1, 3, 6, and 12 months (M). The VISA-P is illustrated in **A** PS group, **B** PE group and **C** PS and PE group panels. MBS is illustrated in the **D** PS group, **E** PE group, and **F** PS and PE group panels. PRP + Sham: PS group. PRP + ESWT: PE group. The *p-value < 0.05, **p-value < 0.01 are as compared with the data before treatment (Pre)

The VISA-P was used to assess symptoms, simple tests of function, and the ability to play sports in the athletes. Both the PS and PE groups showed significant improvements in VISA-P scores at the 1, 3, 6, and 12-month follow-ups after treatments (Fig. 3A, B). There were no significant differences between the two groups at any timepoint (Fig. 3C).

The MBS was used to classify the symptoms of jumper’s knee in athletes. After treatments, both the PS and PE groups showed significantly reduced scores at the 1, 3, 6, and 12-month follow-ups (Fig. 3D, E). There were no significant differences between the two groups at any timepoint (Fig. 3F).

### The changes of cytokines and VEGF-A before and after treatments in the patellar tendinopathy of athletes

Serum levels of IL-6, IL-15, IL-33, IL-17, and VEGF-A were measured using the ELISA assay (Table 3). Differences in serum levels of IL-6, IL-15, IL-33, and IL-17 before and after treatments were observed in both groups, with IL-33 showing no significant difference at the 12-month follow-up (Table 3). Levels of IL-6, IL-15, and IL-17 increased at the 12-month follow-up; however, athletes did not experience any discomfort or injuries detectable by ultrasonography after the study (Tables 3 and 4). The level of VEGF-A only had significant difference compared with pre-treatment status in the PS group at final follow-up (Table 3).

**Table 3:** Changes of cytokines before and after treatments

	PS (PRP + SHAM)	PE (PRP + ESWT)	*p*-value*
IL-33			
Before treatment	1.44 ± 0.5	0.69 ± 0.24	> 0.05
After treatment	4.61 ± 1.67	1.70 ± 0.68	> 0.05
*p*-value^#^	> 0.05	> 0.05	
IL-17			
Before treatment	0.06 ± 0.02	0.09 ± 0.02	> 0.05
After treatment	0.15 ± 0.04	0.14 ± 0.03	> 0.05
*p*-value	< 0.05	< 0.05	
IL-6			
Before treatment	1.15 ± 0.13	1.71 ± 0.39	> 0.05
After treatment	1.97 ± 0.34	1.32 ± 0.25	0.031
*p*-value	< 0.05	> 0.05	
IL-15			
Before treatment	19.07 ± 7.32	7.70 ± 1.41	> 0.05
After treatment	19.22 ± 6.02	11.15 ± 1.47	> 0.05
*p*-value	> 0.05	< 0.05	
VEGF-A			
Before treatment	244.70 ± 45.47	218.00 ± 33.74	> 0.05
After treatment	220.55 ± 44.54	226.04 ± 34.97	> 0.05
*p*-value	< 0.05	> 0.05	

*The *p*-values less than 0.05 are accepted as statistically significant as compared between the PS and PE groups

^#^The *p*-values less than 0.05 were accepted as statistically significant as compared with the data before treatment (Pre)

**Table 4:** Results of ultrasonographic examination

	PS (PRP + SHAM)	PE (PRP + ESWT)	*p*-value*
Distal thickness			
Before treatment	5.01 ± 2.12	5.66 ± 2.28	> 0.05
After treatment	5.03 ± 1.67	5.29 ± 1.54	> 0.05
*p*-value^#^	> 0.05	> 0.05	
Distal elasticity			
Before treatment	61.41 ± 13.67	56.48 ± 12.08	> 0.05
After treatment	68.59 ± 19.75	59. 17 ± 14.83	> 0.05
*p*-value	> 0.05	> 0.05	
Proximal thickness			
Before treatment	5.13 ± 1.38	5.74 ± 1.77	> 0.05
After treatment	5.35 ± 1.53	6.29 ± 2.10	> 0.05
*p*-value	> 0.05	> 0.05	
Proximal elasticity			
Before treatment	63.57 ± 20.95	57.74 ± 16.25	> 0.05
After treatment	66.70 ± 18.21	62.70 ± 18.89	> 0.05
*p*-value	> 0.05	> 0.05	

The *p-value < 0.05 is as compared between the PS and PE groups

The ^#^*p*-value < 0.05 is as compared with the data before treatment (Pre)

### The analysis of ultrasonography after treatments

The details of the ultrasonographic results are presented in Table 4. There were no discernible differences in any ultrasonographic parameters within either group before and after treatment. Additionally, no differences were found between the two groups in any parameters.

## Discussion

PRP has been widely used in recent years because of its high concentration of growth factors. Dr. Dragoo randomized 23 patients into a PRP group or a dry needling (DN) group. The results showed that leukocyte-rich PRP injection accelerated improvement in function (VISA-P) at 12 weeks, but this benefit dissipated over time by 26 weeks [18]. Because the efficacy of PRP treatment for chronic patellar tendinopathy diminishes over time following a single session, some studies have reported that multiple PRP injections can provide more satisfactory long-term outcomes and faster symptom relief [17, 24, 25]. However, there have been varying results regarding PRP treatment for patellar tendinopathy. Dr. Scott compared the efficacy of leukocyte-rich PRP injection and leukocyte-poor PRP injection with normal saline (NS) injection for chronic patellar tendinopathy. In their study, no remarkable improvement was noted in VISA-P and numerical pain rating scale (NRS) scores at any of the follow-up visits [26].

In our experiments, 1 month after PRP injection and combined with ESWT, VAS and functional scores improved significantly. We allowed the athletes to resume training and competitive games throughout the follow-up period. Under these circumstances, most athletes returned to training as soon as their clinical condition permitted and achieved good results. The trend of VAS and functional improvement was maintained until the last visit. Despite the pain relief and functional improvement observed in athletes with chronic patellar tendinopathy treated with PRP alone and combined with ESWT, our 1-year follow-up period did not allow for observation of midterm or long-term therapeutic effects. Due to the potential dissipating effect of a single PRP injection, we are interested in exploring the combined therapy of PRP and multiple ESWT applications for chronic patellar tendinopathy.

In the existing literature, the therapeutic effects of PRP injections typically last around 6 months [17, 18]. However, our study demonstrated that these effects persisted for a longer duration, lasting up to 1 year following a single PRP injection. We believe this prolonged effect can be attributed to several key factors. First, regarding participant demographics, the athletes in our study were relatively young, primarily consisting of high school team members. This younger demographic may enhance recovery and responsiveness to treatment. Second, in terms of collaborative communication, the coaches of the participating athletes maintained close communication with our medical team, enabling real-time adjustments to the training intensity of athletes and frequency following treatment. This tailored approach may have contributed to more effective management of the condition post-injection. Third, we hypothesize that the integration of ESWT may play a significant role in prolonging the therapeutic effects of PRP. By effectively addressing the underlying pathology, the combination treatment may reduce the need for multiple PRP injections, thereby extending the duration of symptom relief. Despite these positive outcomes, it is important to note that out of the 33 athletes in our study, five experienced residual symptoms and required additional treatment after 1 year of follow-up. This indicates that while the treatment was generally effective, there remains variability in individual responses that warrants further investigation.

ESWT, generating by a high-voltage condenser spark discharge, is a high-amplitude sound and focused at targeted lesion through an elliptical reflector [27]. ESWT had been applied in musculoskeletal disorders for more than three decades. ESWT had attracted attention and interest because of its therapeutic effect including antiinflammation, cell proliferation, and neovascularization [27]. ESWT had positive effect in relieving pain and restoring function of patients with tendinopathies of the shoulder, elbow, and heel [28–31]. One randomized controlled clinical trial conducted by Dr. Wang compared the effectiveness of focused ESWT with conservative treatments, including nonsteroidal antiinflammatory drugs, physiotherapy, exercise programs, and the use of knee straps, in patients with chronic patellar tendinopathy [13]. The results showed that focused ESWT is more effective than traditional conservative treatments. However, some studies have reported no effect of ESWT on patellar tendinopathy [16, 32]. Despite existing controversy regarding the effect of ESWT on chronic patellar tendinopathy, a recent systematic review concluded that ESWT is a promising and safe therapeutic modality [33].

Most cases of patellar tendinopathy can resolve with conservative treatment. However, for patients who do not respond to conservative measures, PRP and ESWT are used as alternative options. Dr. Vetrano randomized 46 patients with chronic tendinopathy into PRP and ESWT groups. In their single-blind randomized clinical trial, the PRP group showed significantly greater improvements in VAS, VISA-P, and MBS scores compared to the ESWT group at the 1-year follow-up [34].

There are limited data on the combined use of PRP and ESWT in patients with chronic patellar tendinopathy. In our study, ESWT did not provide additional benefits for chronic patellar tendinopathy treated with PRP injection. There were no significant differences between the PS and PE groups in VAS, VISA-P, and MBS scores at the final follow-up visit. However, the combination of PRP and ESWT accelerated pain relief in these athletes. At our 1-month follow-up, the combined PRP and ESWT group showed greater improvement in VAS scores compared with the PRP injection alone group (Fig. 2C).

The etiology and pathogenesis of tendinopathy are multifactorial, and its biomechanism remains unanswered. Molecular inflammation plays a crucial role in tendinopathy, where complex immunological interactions can lead to the transition from a healing response to chronic symptomatic disease in tendons [35]. In our study, the difference of serum levels of IL-6, IL-15, IL-33, and IL-17 are noted before and after serum biomarkers can reflect systemic inflammatory responses, metabolic changes, and tissue remodeling associated with tendinopathy. Biomarkers such as cytokines, growth factors, and matrix metalloproteinases (MMPs) have been studied in tendinopathies, as they are involved in tissue degradation, inflammation, and repair mechanisms. In particular, increased levels of proinflammatory cytokines and matrix-degrading enzymes have been linked to tendon pathology, and their presence in serum could indicate ongoing tendinous changes or responses to treatment. Furthermore, treatment modalities such as ESWT and PRP are known to modulate inflammatory and healing pathways, which could be reflected in serum biomarkers. In the treatments of both groups, IL33 showed no significant difference at 12-month follow-up (Table 3). IL6, IL15, and IL17 were increased at 12-month follow-up but the athletes did not feel any discomfort or injury by ultrasonography after the study (Tables 3 and 4). We hypothesized that the observed increase in IL-6, IL-15, and IL-17 levels is closely correlated with athletes returning to training after experiencing symptom relief. VEGF is one of the important growth factors for angiogenesis. Chronic patellar tendinopathy had increased cellularity and vascularity and disruption of tissue architecture in pathological histology [36]. In the present study, the level of VEGF-A only had significant difference compared with pre-treatment status in the PS group at final follow-up (Table 3). There are no significant differences in PE group or between groups. However, we cannot make a firm conclusion and explanation for VEGF-A change in this study for some reasons. Furthermore, we suggest that ESWT plays an inhibitory role in the inflammatory response caused by PRP injection, contributing to greater pain relief in the PE group at the 1-month follow-up (Fig. 2c). We believe that both PRP injection and ESWT induce a remodeling healing process in chronic patellar tendinopathy.

Chronic patellar tendinopathy, which caused by repetitive stretching, microinjury, and chronic inflammation, had altered mechanical and morphological tendon properties. Stiffer and larger tendon on painful area of patellar tendinopathy has been reported [37]. Dr. Wiesinger reported that larger cross-sectional area and lower stiffness were noted in tendinopathic patients [38]. Dr. Wang mentioned that a significant increase in vascularity and decrease in thickness of the patellar tendon were noted after shockwave treatment [13]. However, in our study, there were no significant changes in thickness and elasticity observed after treatment in either group, even though athletes resumed training and competitive games throughout the follow-up period.

Limitations exist in the present study. Firstly, the sample sizes are relatively small, despite this being a double-blind randomized clinical trial that meets statistical requirements in power analysis. Secondly, there is no standardized protocol for the use of PRP and ESWT in chronic patellar tendinopathy, and further experiments are needed to establish one. Multiple injections of PRP for chronic patellar tendinopathy have been reported with good clinical outcomes [20, 24], while protocols involving two sessions of focused ESWT and multiple sessions of radial ESWT have also been documented [39]. This single-center trial, with a sample of 33 participants, provided adequate statistical power to detect significant differences in the primary outcome. However, a larger sample size might have enhanced the precision of secondary outcome measurements. Additionally, while we conducted a 1-year follow-up, chronic patellar tendinopathy can fluctuate over time. Longer follow-up period beyond 1 year would offer better insight into the long-term sustainability of the treatment effects.

## Conclusions

Autologous PRP injection and combination with ESWT are both effective treatments for chronic patellar tendinopathy in athletes. We demonstrated significant improvements in pain and functional scores following PRP and PRP with ESWT treatments. This study represents the first analysis of the therapeutic effects of PRP and combined PRP injection with ESWT for chronic patellar tendinopathy. Our results indicate that combined treatment could facilitate earlier pain relief compared with PRP alone and is a safe treatment modality. No adverse effects were noted in our study.

## Data Availability

All data relevant to the study are included in the article or are available as supplementary files.

## Abbreviations

PRP: Platelet-rich plasma
ESWT: Extracorporeal shockwave therapy
PS: PRP + Sham
PE: PRP + ESWT
NSAIDs: Non-steroidal antiinflammatory drugs
VAS: Visual analogue scale
VISA-P: Victorian Institute of Sport Assessment-Patella
MBS: Modified Blazina scale
ELISA: Enzyme-linked immunosorbent assay
VEGF-A: Vascular Endothelial Growth Factor-A
IL: Interleukin
FDR: False discovery rate
DN: Dry needling
NRS: Numerical pain rating scale
